# Textronic Solutions Used to Produce Layers Sensitive to Chemical Stimuli—Gas Sensors: A Review

**DOI:** 10.3390/ma16175893

**Published:** 2023-08-29

**Authors:** Ewa Skrzetuska, Paulina Szablewska

**Affiliations:** Faculty of Material Technologies and Textile Design, Institute of Material Science of Textiles and Polymer Composites, Lodz University of Technology, 116 Żeromskiego Street, 90-924 Lodz, Poland; paulina.szablewska@dokt.p.lodz.pl

**Keywords:** chemical sensor, gas sensor, textronics

## Abstract

Thanks to the intensive development of textronics, textronic applications are already visible in many areas of everyday life. Many researchers around the world have focused on the invention of textronic systems to increase security, create technological innovations and make everyday life easier and more interesting. Due to the wide use of chemical textile sensors, this review article lists scientific publications covering all types of wearable chemical sensors along with their latest developments. The latest developments from the last few years in moisture, pH, sweat and biomolecules sensors are described. In this review, greatest emphasis and detail was placed on textile gas sensors and their production methods. The use of, among others, graphene and zinc oxide grown on cotton fabric, colorimetric textiles based on halochromic dye, electronic graphene fabric based on lotus fibers and graphene oxide and zinc oxide nanorods were considered. Finally, this article summarizes our current knowledge on gas sensors, compares the detection properties of the presented projects and indicates future directions of development.

## 1. Introduction

Times of dynamic technological development have resulted in research work on minimizing electronic systems. As early as the 20th century, the term and idea of textronics were introduced to connect electronic circuits with textiles, as in 1980, Steve Mann was integrating many textronic components into clothing. Thus, scientists around the world began to focus on the development of this field [1]. Many types of textronic sensors have been invented, responding to stimuli of various sources ranging from physical, biological and chemical ones. Their response can be programmed and built in many ways, including changes in resistance, impedance and color change. Textronics are present in many fields of life. There are applications in medicine, sport, health and safety, motorization, military, entertainment, heating materials, communication and fashion. They provide a lot of valuable information about one’s state of health, target parameters or delight with their design. The topic of connecting electronic systems and textiles is new, and many innovative solutions and applications are to be discovered. The field is rapidly developing due to the high demand for smart clothing in all areas of everyday existence. There are already projects that try to combine several areas of life into one solution. A group of researchers led by E. Saoutieff developed a product in the form of a sensor platform. The product is characterized by low power consumption, a wireless design and energy efficiency and has a multifunctional wearable system. The project offers one the ability to monitor their lifestyle, obtain real-time healthcare and collect medical data. The system is compatible with several types of sensors. The goal was to combine the platform with other products that can monitor not only a person’s health indicators but also the external environment. The analysis will involve the chemical molecules NOx, COx and NHx to monitor health exposure on an ongoing basis [2]. 

The sensors considered in this article are products with a textile base. Chemical sensors sensitive to various analytes are significant in their current existence, but the greatest emphasis is to be placed on the sensors that provide the user with protection against the harmful effects of the external environment and to monitor their health condition. Focusing on safety and protection during everyday activities such as cooking on a gas stove or in between these activities on the gas installation itself, at the end of 2022, during the heating season, firefighters in Poland recorded 51 carbon monoxide interventions in one week residential buildings. Unfortunately, because of carbon monoxide exposure, seven people lost their lives and twenty-two suffered from poisoning. From the beginning of the heating season, from 1 October 2022 to 1 December 2022, firefighters recorded 589 interventions, and as many as 23 people lost their lives and 184 were poisoned [3]. Even though fireplaces and gas installations are still actively used in many Polish homes and citizens are trained in preventing tragic accidents related to a gas leaks at home at every step of gas installation, statistically only one in fifteen Poles has a gas or smoke detector at home. Poles appear to not see the need to install such devices, and they justify this with their lack of time and lack of legal regulations on this subject. In addition, the public admits that they do not know how to use this type of sensor and therefore do not need this type of equipment. Moreover, the aesthetics of such devices do not encourage individuals to hang them indoors. Despite the excellent parameters and quick response of gas devices, they are too big and nonattractive for the eye [4]. 

The aim of this paper is to review the existing knowledge in the field of all types of textile chemical sensors to present an increasing number of applications of sensors in many areas of life, to show the solutions of researchers around the world who used many varied materials, to show the methods of fabrication and focus on textile gas sensors in detail. Regarding textile gas sensors, the current state of knowledge and shortcomings that may be overcome in the future will be described.

## 2. Textile Chemical Sensors

**Figure 1:**
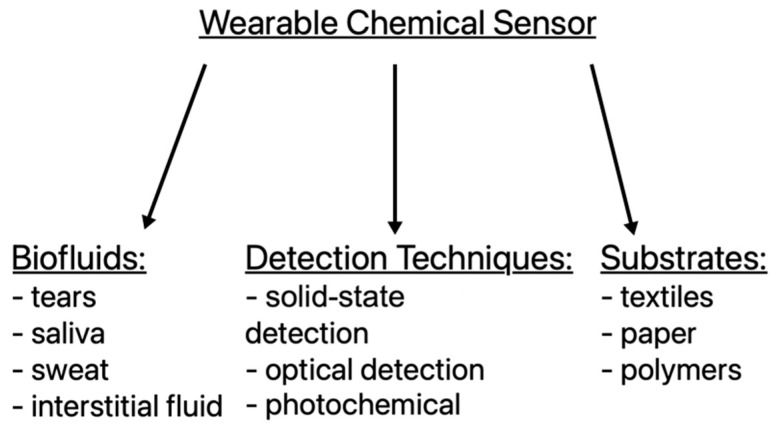
Division of wearable textile chemical sensors.

Wearable chemical sensors are divided into two subgroups according to their detection technique—electrochemical and optical. They can have different bases like textiles, polymer hydrogels or paper and detect an entire range of chemically active analytes [5]. The development of wearable chemical sensors has been gaining momentum for years due to their many applications. Solutions can be implemented in areas such as medicine, safety, health and sports [6]. Sensors in this group are based on changes in conductometry. Results can be monitored in real time by changes in electrical conductivity [7], resistivity or color [8]. There are many types of textile chemical sensors, such as humidity, sweat, pH, gas or biomolecule sensors [1]. They use a wide range of materials and active substances. The division is shown in Figure 1. The choice of these two components is an important aspect in creating wearable electronics. Together, they must meet the requirements of electrical performance, mechanical strength, flexibility, durability, and lightness, and in addition, their production is cheap. It is also impossible to consider the functional properties that are supposed to ensure comfort of use, i.e., air permeability, moisture and aesthetic values [9]. There are several groups of active substances: conductive polymers [10], carbon-based materials on the micro- and nanoscale [11] and conductive metallic structures on the nanoscale [12].

Minimalism in textronics is one of the main goals of textronic development. The aim is to achieve solutions in which all electronic functions could be implemented in the fiber itself. Such fibers would allow textronics to be integrated with the textiles already in place during the weaving process. Currently, for technological reasons, the production of such a product is very difficult. Polymers are the materials of the future, especially organic polymers, and they are low-molecular-weight compounds. They are characterized by flexibility and integrate into fiber-producing composite materials. As they are made of basic building blocks, it is possible to adjust their chemical, physical and electronic properties to meet the required needs [13]. Organic polymers exhibit electrical properties like those of metals. Examples of conductive polymers are polypyrrole, polyaniline and polythiophene. Organic polymers are the most promising materials [14]. There are 0-, 1- and 2-dimensional carbon-based micro/nanomaterials used in textronics—graphene, in the form of nanosheets, and carbon nanotubes and nanoparticles. They are flexible and suitable for use in textronic systems. They are characterized by high conductivity, impeccable mechanical properties, a huge surface area, high stability and low production costs. The most-used nanomaterials in research are carbon nanotubes and graphene. Metal nanostructures are great for making flexible and wearable fiber-based electronics because they boast remarkably high conductivity. They have small dimensions; examples are nanowires, nanorods and nanoparticles. The known metals used are silver, zinc oxide, gold and platinum. They are also available in such forms as pastes, whereby they obtain excellent conductive properties. The downside of this form is the demanding stability. Another form is thread. Metal threads can be a component factor of the material and can be a part of it or be embroidered in the form of embroidery already at the weaving level. They have conductive properties and are resistant to sweat and washing. Metal nanostructures can also be applied to textiles in the form of a thin film [1,6]. Metal oxides, carbon nanotubes, conductive polymers and 2D materials are classified as semiconductors; hence, the detection solutions made of them are called semiconductor gas sensors. The principle of operation of these sensors is the direct reaction of gas molecules with the sensing material, changing its parameters such as its conductivity, permittivity and work function. Then, the transducer present in the system converts the received information in the form of physical parameters into electrical parameters, such as resistance, capacitance and inductance. It successively produces a voltage or current signal characterized by measurable magnitudes such as frequency and phases. Semiconductor gas sensors, where there is a direct interaction between the gas molecules and the treatment material, allow for the definition of characteristic values such as the sensitivity, stability and biocompatibility [15]. Currently used on a large scale, three subgroups of semiconductor gas sensors can be distinguished: electrochemical sensors, which are based on electrolytes; pellistors based on catalytic combustion; and conductometric/chemoresistance-based gas sensors, which are based on the resistance modulation of semiconducting oxides [16].

**Figure 2:**
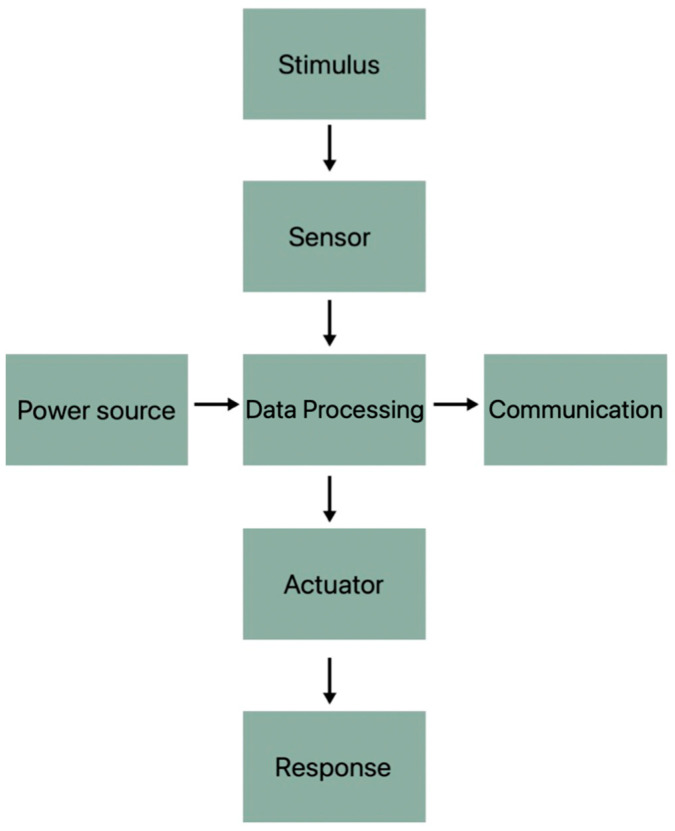
Block diagram of the cells needed for the proper operation of textile sensors.

Figure 2 shows the mandatory components of the textronic system and the order in which they must be placed next to each other. The sensor whose task is to detect the intended stimulus needs, depending on the operating technique, a power source, an element that converts the stimulus into a signal, an analyzing cell and a response element.

## 3. Humidity, Sweat, pH and Biomolecules Sensors

Moisture-sensing sensors are used in medical solutions for wound care. In the case of this type of sensor, it must have appropriate electrical conductivity and sensitivity to water molecules. Zhou et al. developed a textile-based moisture sensor together with Single Walled Carbon Nanotube/Poly(vinyl alcohol) fibers in a wet-spinning process. They produced ultrastrong fibers on cotton fabric to show that the humidity sensor could monitor human sweating. Finally, the sensor was shown to monitor human breathing before and after exercise to analyze water molecules, which are an essential component of breathing, and which affect the relative humidity (RH) around the mouth and nose [17]. 

Ma et al. developed a significant sensitive and responsive humidity sensor. Yarn can be embedded in wearable devices to detect human breath. The pandemic period has shown the importance of personal protective equipment and has created a demand for disposable and reusable products for this purpose—masks. The biaxial yarn was wrapped twice on a copper wire and then stitched onto a face mask to monitor human breathing in real time. Another study developed a fiber capacitive moisture sensor. The composite yarn based on a silk-wrapped copper wire was brushed with polyimide and then sputter coated with silver to produce a moisture sensor. Researchers placed the developed sensor in a smart face mask and showed that the sensor can monitor the wearer’s breathing conditions. The accurate and continuous real-time monitoring of vital signs such as respiratory rate by using electrochemical sensors on face masks can help analyze and evaluate patients’ health status, establish diagnoses and predict recovery [18]. 

Sakandar Rauf et al. developed a smart textile sensor for humidity detection by using a metal−organic framework (MOF) as an active thin-film layer. Researchers used the Langmuir−Blodgett (LB) technique of depositing an MIL-96(Al) MOF in a thin layer directly onto fabrics containing alternating textile electrodes to fabricate a highly selective moisture sensor. These sensors are made of two diverse types of textiles—linen and cotton. The sensors showed an elevated level of selectivity in detecting water vapor in the presence of several volatile organic compounds (VOCs) [19]. 

Su et al. proposed a sensor-transduction-coupled strategy. They exploited the embedding of high piezoresponse Sm-PMN-PT ceramics in a moisture-sensitive polyetherimide polymer matrix by electrospinning. The aim of this treatment was the synchronous and sympathetic coupling of moisture perception and signal transduction. They revealed through a phase-field simulation, experimental characterization, the design principle of the composition and the topological structure of the piezoelectric sensing–transducing-coupled textiles that the textiles can modulate the recognition rate, conversion and use of the sensitive components of the prepared active humidity sensors, achieving high sensitivity and a fast response to ambient humidity. The prepared fabric made of piezoelectric-coupled textiles can be worn on the human body to realize emotion recognition, exercise status monitoring and physiological stress identification [20]. 

He et al. described ultrafast, sensitive, textile-based moisture sensors made of single-walled carbon nanotubes, polyvinyl alcohol and lithium chloride nanocomposite fibers produced by a wet-spinning and solvent-exchange process. The produced humidity sensors showed high stability during deformation and high sensitivity to significant resistance changes in a wide range of relative humidity conditions. The so-called ultrafast sensor responses were the result of LiCl rapidly deliquescing upon contact with water molecules and generating ions that caused a change in conductivity. Textile moisture sensors responded correctly to human breathing and changes in humidity in real time. These phenomena made it possible to distinguish between different respirations and changes in the microclimate. Their features of high detection performance and their flexibility allow them to be combined with textiles and qualify for wearable sensors [21]. 

Chen et al. first proposed a stretchy-paper origami moisture sensor compatible with textiles. Even though paper itself does not have such stretchability, this sensor can achieve good results. This is due to the integration of origami folding structures into a paper substrate, where air-laid paper acts as both a sensing material and a sensor substrate. The sensor produced by the researchers has high sensitivity, a correct response and recovery properties with excellent stability during deformation. Performance tests were carried out after 300 cycles of folding and unfolding, which showed its ability to monitor the respiratory rate continuously and dynamically. Features such as flexibility and extensibility allow the textile sensor to be combined and used for respiratory rate monitoring and diaper-wetness detection [22]. 

The current topic is the COVID-19 pandemic, which for some time made people around the world live in protective masks. Scientists from Serbia have developed innovative disposable masks with a humidity sensor. The humidity sensor consists of alternating electrodes made of conductive threads based on polyamide and ordinary polyester threads, which act as a dielectric sensing layer embroidered between them. As a result, the sewn-in sensor has features similar to a capacitor. During the tests, the moisture adsorbed by sensitive hygroscopic polyester threads changed their dielectric properties and permeability. This was detected by changing the capacitance values of the sensors at various levels of relative humidity. The produced sensors showed repeatability and stability in various humidity conditions for 80 min [23]. 

Staying on the topic of masks, the team of scientists led by P. Kumar created an antibacterial fabric with photothermal disinfection properties. The work uses MoS_2_ modifications to produce polycotton fabrics with brilliant antibacterial activity and photothermal properties, and so the possibility of adding an active function to protective masks was proposed. From the conducted experiments, the researchers proved that after irradiating a sample of the modified fabric with sunlight, its surface temperature increased to ~77 °C. The given temperature allows for the self-disinfection of the fabric. A valuable piece of information is the fact that complete self-disinfection takes place 3 min after the start of insolation. The product is resistant to washing—scientists tested the self-disinfecting properties even after 60 cycles and the efficiency was 98% compared to the original state. Due to the potential use of a fabric modified by MoS_2_ nanoparticles, its functional properties should be examined. It has been proven that after applying a layer of fabric, this treatment did not change the breathability parameter of the final product. Turning to the numbers, the surgical mask with the layer of modified fabric showed a 97.06 ± 0.37% filtration efficiency for 200 nm particles and a 96.18 ± 0.92% efficiency for 100 nm particles. In comparison, the values for a normal surgical mask are 90.55 ± 2.15% for 200 nm particles and 86.94 ± 1.28% for 100 nm nanoparticles [24].

Researchers led by Dr. Hasanpour presented their achievements in their research on a multistage roll-to-roll dip-coating method. The method aimed to produce meters of cotton threads impregnated with nanoparticles with a coating of fluorinated ethylene and propylene (FEP). The purpose of using the FEP coating was to filter out the interference of the obtained signal of dependence of the temperature readings on humidity. Scientists have built a sensor that combines the ability to measure two quantities—temperature and humidity. It is capable of quickly measuring relative humidity in the range of 30–80% and temperature changes in the range of 20–90 °C. It is also characterized by durability up to 6 months. To test the sensor in practice and verify its correct operation in medical-wound-monitoring applications, it was integrated with a silicone-based dressing. The experiment showed no negative effect on the cells [25]. 

Liu et al., by using the chemical click method, i.e., atomic–economic reactions commonly used to connect two molecular units of their choice, made hydrophobic–hydrophilic patterns. They were built on the surface of a knitted fabric containing polyester and spandex. Patterns made of silver nanowires were applied only in the area where the fabric has hydrophilic features. The purpose of this procedure was to ensure accurate and repeatable motion-detection signals. The resulting sensor recorded a low electrical resistance of less than 60 Ω, a stable resistance cyclic response of more than 2000 cycles and a fast response time to humidity of 0.46 s during the detection evaluation. Using the same method, the researchers made a sensor made of carboxylic carbon nanotubes previously modified with polydopamine. During tests, it showed high sensitivity. Not only was the laboratory-regulated ambient humidity tested, but also breath and skin humidity during everyday existence [26]. 

Sweat sensors, like those that analyze the secretions of the human body, must be in contact with the skin. They must be in clothing or accessories that adhere to the body. It can be clothing like a T-shirt or socks, a material band or underwear. The scheme of operation of sweat sensors is based on the flow of the current and the accompanying parameter, which is resistance. Sweat monitoring can provide valuable information about hydration, pH and changes in ions such as K^+^ and Na^+^. Analyzing human physiological fluids allows one to control their health and detect diseases earlier. Sweat is a bioliquid that is easy to collect in a noninvasive way. Schazmann et al. created a textile sensor built into a belt so that the sensor is in contact with the skin and analyzes the Na^+^ ions in sweat in real time. The strip containing the sensor is made of a sodium-ion-selective electrode. This product is used in patients suffering from cystic fibrosis [27]. 

Other methods of analyzing sweat in textile sensors are the optoelectronic probe and colorimetric sensor. Shirley Coyle et al. first announced the implementation of an optoelectronic probe embedded in textiles. The solution was based on a photometer. The screen-printing production method was used to apply a hydrophobic acrylic paste on both sides of the material. The researchers made a hole in one end through which sweat could enter the analyzing sensor. They used capillary flow here. For pH analysis, scientists implemented bromocresol violet as a sensitive dye. It is adequate because it changes its color from yellow to blue across the pH range. A paired emitter–detector LED was also installed to quantify the color change in the dye. The prototype of the device showed proper operation during practical tests on the body during physical exercise [28]. 

Other scientists using a textile colorimetric sensor were N. Promphet et al., whose task was to detect lactate and the pH of sweat. During the tests, three different layers—chitosan, sodium carboxymethyl cellulose and an indicator matrix—were deposited on a cotton fabric. Researchers used a mixture of two organic dyes—methyl orange and bromocresol green—to analyze the pH. Across the pH range, the color changed from red to blue, while the intensity of the violet color was increased by increasing the sweat lactate concentration [29].

I. Gualandi et al. developed a chemical sensor for the analysis of sweat based on conductive polymers. It has a lot of advantages—excellent mechanical properties, electrical conductivity comparable to semiconductors and conductive polymers that are biocompatible. After appropriate fabrication, it is possible to measure pH, ions, glucose and lactate on a multisensing platform. Research is also underway to detect other analytes. These include adrenaline and dopamine [4]. 

L. Possanzini et al. developed a textile, multithreaded biosensor platform. It can detect different analytes without visible interference. It has been tested for the detection of chloride ion concentration and pH. Structurally, the sensor consists of synthetic and natural threads coated with a conductive polymer—PEDOT: PSS—and functionalized with a composite material and a dye sensitive to a chemical stimulus. Single-thread sensors are characterized by high sensitivity, repeatability, stability and selectivity and can analyze the environment even at low concentrations of solutions [30].

Ha et al. proposed an original solution of a colorimetric sweat-pH sensor based on electrospun turmeric fibers and thermoplastic polyurethane (C-TPU) for diagnosing disease states. By separating the H atoms, the sensor changes color in response to changes in the chemical structure of the sweat’s pH. Researchers also found that with O_2_ plasma activation and thermal pressing, it is possible to bond the sensor to textiles [31]. 

Kim et al. presented a miniaturized system solution based on microfluidic technology. This sensor quickly calorimetrically assesses the concentration of nutrients in sweat such as vitamin C, calcium, zinc and iron. A transdermal patch is present in the system. Researchers compared the correct operation of the built system with traditional measurement methods. The results showed high accuracy [32]. 

Biomolecular sensors are at the forefront of sports applications. They are used for the analysis of proteins, nucleic acids and small molecule drugs. However, there is no shortage of solutions for the medical industry. K. Nomura et al. produced a textile blood-leak sensor on cotton fabric. They combined two novel techniques. The first was the screen-printing technique, which applied two or three layers of conductive ink paste on a silicone blanket. It allowed one to avoid undesirable blurring, short circuiting between adjacent conductive patterns and the breakage of the electrode. An important aspect is the development of a scheme for distinguishing blood from other substances. To this end, the researchers used a second technique based on the specific dielectric dispersion of blood observed in the submegahertz frequency range. The reference substance was human sweat. The sensor is intended for the detection of blood leakage during hemodialysis [33]. 

Ma et al. produced a film-based biosensor that monitors bioparticles in real time. It consists of a reduced layer of graphene oxide and Prussian blue. The sensor was fabricated by using electrostatic interactions at the oil/water interface driven by minimizing interfacial free energy. The prepared sensor can be easily woven into fabrics and treated as a wearable sensor. The biosensor shows high glucose-detection capabilities and is characterized by its mechanical strength during movements such as bending and twisting. The sensor can connect to a smartphone and detect biomarkers in real time [34]. 

## 4. Gas Sensors

Diverse types of gases, even harmful ones, accompany people every day. They are present at home, in the workplace or on the street. Due to continuous and intensive technological development, changes in the energy sector of many countries and the growing percentage of cars, the concentration of harmful gases in the air is constantly growing. However, to minimize the adverse impact on health, many innovative solutions are introduced. Hybrid and electric cars, photovoltaics, heat pumps and electrical installations in the form of, for example, induction hobs instead of gas installations are commonly used nowadays [35,36,37]. Despite the replacement of gas installations with other solutions based on electricity, they are still present in many homes. They are used to prepare meals and to heat water for daily hygiene or heating one’s apartment. The risk of using a gas installation is high. In extreme cases, a leak or explosion can lead to death. As mentioned in the introduction, a small number of residents of houses and flats can boast of the presence of a gas detector in their home, but this amazing number is due to the interventions of special services, and people can become injured or victims because of a leak or explosion of their gas installation. Also, the gas installations present in vehicles are associated with high risks of use. In such small spaces, it is difficult to install a gas sensor as it is quite a large device. Since there are currently many minimalist solutions for monitoring human vital signs, air quality and many other important parameters in the form of watches, bands or other small devices, it would be necessary to create an attractive solution for monitoring harmful gases in the presence of the user, which has a significant impact on health and life. Many researchers around the world are interested in this topic, and many solutions for textile gas sensors have already been proposed, but they are still not publicly available. 

The following raw materials are used to produce wearable sensors monitoring the environment: for substrates: polymers, silicone elastomers, gels and cellulose nanofibers; for the active layer and electrodes: metals, allotropic forms of carbon, polymers and nanocomposites. Various production methods are used: coating, printing, physical vapor deposition, chemical vapor deposition, in situ growth, embroidery, laser-induced forward transfer and lithography [38,39]. Gas sensors can come in various forms depending on the intended use, such as textiles in headbands, rings, tattoos, tags, gloves and masks [40]. Due to the specification of use, such a device must be characterized as small sized, low weigh, having low energy use, ecofriendly, having biocompatibility, being easy to integrate with components and having immense resistance to mechanical deformation. An important and the hardest procedure to perform is to produce the detector which, under the influence of mechanical forces such as tensile force or bending, will provide a clear signal without interference and noise [41].

Gases that are harmful to humans and threatening to one’s health or life are, among others, carbon dioxide, carbon monoxide, sulfur dioxide, ammonia, chlorine, hydrogen chloride, hydrogen sulfide, nitrogen oxides, nitrogen dioxide and propane–butane. 

Carbon dioxide (CO_2_) is a colorless and odorless gas, a natural part of the air. In recent decades, the concentration of carbon dioxide in the atmosphere has increased significantly, and since the late 1990s, the concentration of CO_2_ in the Earth’s atmosphere has increased by almost 25%. Carbon dioxide is produced during the combustion of solid and liquid fuels, plant and animal remains and waste. It is also a natural product of the respiration of living organisms, bacteria, plants, humans and animals. It is formed during composting as well as in putrefactive processes. In addition, it is secreted by plants when they do not have access to light [42]. Carbon dioxide is commonly known as carbon monoxide. It is a highly toxic substance that causes poisoning. Inhaling carbon dioxide causes carbon monoxide to enter the body through the respiratory system and then enter the bloodstream. Carbon dioxide in the body quickly binds to hemoglobin and other metalloproteins containing iron, which leads to the blockage of oxygen supply. CO_2_ poisoning, or smoke inhalation, causes symptoms that may not be specific and therefore difficult to recognize. General indisposition and headache may occur. These ailments can be dangerous when combined with dizziness, vomiting, nausea, drowsiness and general weakness of the body. Carbon dioxide poisoning can cause various symptoms depending on the concentration of CO_2_ in the air and the concentration of carboxyhemoglobin in the blood. Symptoms of chimney-smoke poisoning include shortness of breath; memory disorders; vision problems; palpitations; lack of feeling in the fingers; mental retardation; and the first symptoms of Parkinsonism, such as muscle tremors and a rigid facial expression. Suffering from carbon dioxide poison may also cause hallucinations and, in severe cases, can lead to brain stem damage; speech disorders; and damage to the heart muscles, kidneys and liver [43]. 

Sulfur dioxide (SO_2_) is a colorless gas with a pungent and suffocating odor. In higher concentrations, it is highly irritating and even poisonous. Due to its bactericidal and fungicidal properties, it is used as a food preservative. It is created, among other reactions, during the decomposition reaction of unstable salts of sulfurous acid (sulfites). The negative impact of SO_2_ in the atmosphere manifests itself not so much in the impact on the greenhouse effect as in the formation of so-called acid rain. Acid rain is bad for both plants and soil. On a farm, sulfur dioxide is produced during the combustion of solid fuels—there is a lot of sulfur in coal, liquid fuels and any waste [43]. Sulfur dioxide has a strong irritating effect on the respiratory system of humans and laboratory animals, leading to obstructive and inflammatory changes. Sulfur dioxide is mutagenic, clastogenic and genotoxic and inhibits DNA synthesis, mitosis and cell growth. There is no evidence that sulfur dioxide is carcinogenic. Based on the results of epidemiological studies, the harmful effects of the compound on female fertility and the birth weight of offspring have been demonstrated. Experimental studies have not confirmed the effect of sulfur dioxide on the ontogenetic development of the organism [44].

Ammonia (NH_3_) is a colorless poisonous gas easily recognizable by its pungent smell. Since nitrogen is a component of proteins, the breakdown of these chemicals leads to the formation of ammonia. All ammonia emissions come from agriculture. There are two main sources of ammonia on a farm: animal excrement (79%), especially when improperly managed, and improper fertilization with urea (21%), of which up to 30% of ammonia can be lost in this way. Particularly harmful, both from the point of view of nitrogen losses and unfavorable ammonia emissions, is improper storage and leaving fertilizers containing nitrogen in the form of ammonium, including organic fertilizers, on the soil surface [42]. Ammonia is absorbed in the respiratory tract (respiratory retention is as much as 92%) through the skin and mucous membranes. It is bound in the body and excreted as urea. A significant amount of ammonia is also excreted unchanged in exhaled air, urine and saliva. The good solubility of ammonia in water means that its effect is limited to the mucous membranes of the eyes and the upper respiratory tract. It is particularly dangerous for the eye’s cornea, causing its clouding, ulceration and puncture, which may lead to a complete loss of vision. When exposed to high concentrations, the irritating effect of ammonia on the lungs can cause swelling and changes in their function. In addition, the evaporation of liquid ammonia in contact with the skin can cause frostbite. The allergenic effect of ammonia has not been found [45].

Chlorine (Cl) is a poisonous yellow–green gas. It is a component of many compounds commonly found in nature, especially salts. Chlorine is used in disinfectants (bleaches) and in water chlorination. Chlorine is a mineral element classified as an electrolyte. The basic role of chlorine in the body is to regulate the pH of body fluids, osmotic pressure, nerve conduction and permeability of cell membranes. In addition, chlorine participates in the process of protein digestion and the production of hydrochloric acid in gastric juice. This element also regulates metabolic changes. Too much chlorine in the body is associated with a salt-rich diet, dehydration, loss of bicarbonate through the digestive tract and kidney disease. It is manifested by disorders of the nervous and muscular system, kidney disorders or hypertension. Some people are allergic to chlorine. It especially affects people using a swimming pool. In turn, drinking chlorinated water can lead to food allergies—nausea, vomiting, diarrhea and abdominal pain. Chlorine poisoning refers to the inhalation of chlorine gas. Symptoms of chlorine poisoning include labored breathing, coughing and shortness of breath. The consequence of chlorine poisoning can be pulmonary edema and even death [46]. 

Hydrogen chloride (HCl) is a colorless gas with a suffocating effect. Hydrochloric acid, i.e., an aqueous solution of gaseous hydrogen chloride, is used in the textile industry; for the manufacture of dyes and medicines; and for the removal of metal oxides, e.g., from iron. It is also used in tanning and sugar production. This acid is also used to produce glucose and other starch products. Pure hydrochloric acid is one of the most important laboratory reagents. Symptoms of acute poisoning caused by gas or hydrochloric acid aerosols are eye pain, lacrimation, redness of the conjunctiva, burning pain in the mucous membrane of the nose and throat and cough. In concentrations exceeding the ceiling values, it can cause spasms of the glottis, laryngeal edema and pulmonary edema [47,48]. Hydrogen chloride is present in fire gases with a pungent suffocating and sour odor. Hydrogen chloride in the form of gas or hydrochloric acid aerosols causes eye pain, lacrimation, redness of the conjunctiva, burning pain in the mucous membrane of the nose and throat and cough. In concentrations exceeding the ceiling values, it can cause glottis spasms; laryngeal edema and pulmonary edema; skin contamination; painful chemical burns; eye contamination; and burns of the eyelids, conjunctiva and cornea leading to blindness, while ingestion causes burns of the mucous membrane, mouth, throat and esophagus as well as abdominal pain and bleeding from the gastrointestinal tract, and circulatory collapse may occur [49].

Hydrogen sulfide (H_2_S) is a colorless gas that is toxic to living organisms (including humans, farm animals and plants). It has a characteristic smell of rotten eggs. On a farm, it may be formed because of anaerobic protein decomposition in closed tanks containing liquid manure; in liquid manure in septic tanks and composting pits when the composting process is carried out without access to the right amount of air; and in chicken coops, cowsheds or stables. The concentration of hydrogen sulfide in closed tanks, e.g., in a septic tank, can be so high that it threatens human life. Hydrogen sulfide is also a byproduct in the biogas production process. In this case, however, it is often removed because it can cause the unfavorable corrosion of the devices in which biogas is burned [42]. The main target organs in acute hydrogen sulfide poisoning are the central nervous system and the lungs. High concentrations of hydrogen sulfide cause paralysis of the respiratory system, cyanosis, shortness of breath and death. After exposure to lower concentrations of hydrogen sulfide, conjunctivitis and painful corneal erosions immediately appear, the nose and throat become irritated and bronchitis appears. Bronchopneumonia and pulmonary edema are common complications. Many cases of neurological and neuropsychological changes have been reported following acute poisoning. Under occupational and repeated-exposure conditions, the primary target organs of hydrogen sulfide are the nose, eyes and respiratory system [50].

Nitrogen oxides (NOx)—this is the common name for the whole group of nitrogen and oxygen compounds. Their harmful impact on the environment occurs after they combine with water and, consequently, form so-called acid rain, as in the case of sulfur dioxide. The main source of nitrogen oxides is the combustion of fossil fuels and biomass. Nitrogen oxides are also the result of ammonia oxidation in the atmosphere, produced during agricultural human activity. It is worth considering how you can determine the number of gases produced on the farm. The possible direct measurement of the emission only makes sense if the emission source is point-like. Such point sources of emission are, e.g., furnaces, livestock buildings or storage places for, e.g., compost, slurry, liquid manure, manure, etc., but even then, the measurement may be difficult. For example, the measurement of gases emitted from a pigsty, barn, stable or other buildings can be easily carried out if all the air leaving the building is focused on the designed ventilation outlets. It is more difficult to measure if the rooms are ventilated; for example, by opening windows. Measuring from diffuse sources, e.g., from a field, is difficult, and therefore, the emission volume of individual gases can be determined based on the constantly developed calculators available, e.g., on the Internet or based on publications made by scholars. When using such sources, it is worth remembering that they are based on certain assumptions and estimates that allow for the determination of the order of magnitude, but not the exact emission from a specific farm [42]. Nitrogen oxides, especially NO_2_, have a negative impact on human health. They primarily irritate the respiratory system, posing a serious threat, especially for people suffering from asthma and chronic obstructive pulmonary disease, contributing to the exacerbation of ailments. The inhalation of air with nitrogen dioxide content can result in attacks of shortness of breath, the irritation of mucous membranes, stabbing in the chest and shortness of breath. In addition, it affects one’s resistance to infections and the development of diseases of the circulatory system as well as cancers—especially breast and lung cancer [51].

Hydrocarbons like propane and butane are combustible gases which, when supplied with the right amount and when ignited, burn, emitting carbon dioxide and water vapor while releasing heat energy [52]. Propane–butane is not harmful to humans when used correctly. Its downside is its flammability. However, there are cases of abuse of this gas that have resulted in death.

Carbon monoxide is a highly poisonous, colorless and odorless gas, slightly lighter than air, which makes it easy to mix and spread. Potential carbon monoxide sources in living spaces include fireplaces, gas water heaters, coal, gas or oil stoves and gas cookers. It arises because of the incomplete combustion of many fuels, including wood, oil, gas, gasoline, kerosene, propane, coal and oil, caused by the lack of an adequate amount of oxygen necessary for complete combustion. The lack of signals that would alert people to the presence of carbon monoxide is a significant factor that contributes to carbon monoxide poisoning. The danger of asphyxiation results from the fact that carbon monoxide is a gas imperceptible to humans. It enters the body through the respiratory system and is then absorbed into the bloodstream. In the human respiratory system, carbon monoxide binds to hemoglobin 210 times faster than oxygen, blocking the oxygen supply to the body. This poses a serious threat to human health and life. It prevents the proper distribution of oxygen in the blood and causes damage to the brain and other internal organs. The consequence of acute poisoning may be irreversible damage to the central nervous system, coronary insufficiency and myocardial infarction or even death [53].

The same risks of encountering dangerous gases while doing housework or going for walks apply to dangerous gases in the workplace. In every country, there are regulations regarding the highest possible concentrations of these substances in the surrounding environment. Table 1 presents the previously described values for selected gases. The data come from the ministerial regulation, which is the document currently in force [54]. Table 2 shows the threshold values of gas perceptibility through the human nose. It is obvious that for the detector to fulfill its task, it must have detection values lower than those given in the table.

A group of researchers from Indonesia and Germany described their research work on a gas sensor based on composites of graphene and zinc oxide grown on cotton fabrics by using wet-solution methods. They made four composite samples that differed in the proportions of the seeded components. Diverse configurations of graphene solutions—0.0125 M, 0.025 M and 0.5 M—were tested. Subsequently, the already-prepared fabrics were modified with zinc oxide seed layers deposited by using the solution method. The solution was prepared by the researchers by dissolving 0.1 M Zn(NO_3_)_2_ × 6H_2_O in 2-propanol and heating the solution to 75 °C with magnetic stirring. After 15 min, 0.1 M triethylamine was added as a sol stabilizer. The fabrics were immersed in the solution for 5 min and then dried. Zinc oxide nanorods were grown by a chemical bath deposition. The sensitivity of the prepared samples was measured by monitoring the electrical resistance in a sealed chamber. The detection response was calculated by dividing the difference in the resistance values before and during the detection from the initial resistance and is expressed in %. The sensor response was tested for CO concentrations in the range of 10–90 ppm at a room temperature of 27 °C. The best of the sensors made was the one in which the ratio of graphene and zinc oxide was 1:1. The reaction and recovery times were 370–435 s and 45–115 s, respectively. It was found that honing ZnO nanorods improves the response and performance of the sensor, and increasing the amount of this compound causes a deterioration in the response. The sensors showed resistance to mechanical factors such as bending. The response of the sensors was tested not only for CO but also for other gases that threaten human life, such as nitric oxide, ammonia, acetone and methanol. The sensor response was 40.26% for carbon monoxide, 5.48% for nitrogen oxide, 1.22% for ammonia, 0.9% for acetone and 0.45% for methanol. A study of the sensor response at higher humidity was performed and showed that the value decreases. For CO detection of 50 ppm, it drops to 27–36% [56].

**Figure 3:**
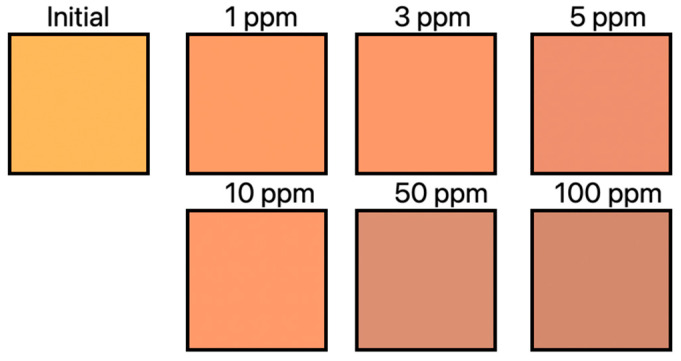
Sensor with Dye 3 color changes depending on NH_3_ gas concentration.

A unique solution that differs from all the previously presented solutions is the colorimetric textile sensor based on the simultaneous detection of NH_3_ and HCl gases. Until now, these textile sensors based on halochromic dyes were produced by electrospinning. Their sensitivity was already at an elevated level, detecting gaseous bases and acids. However, they were also characterized by low durability, low efficiency and high production costs. In one sentence, they have not found their application in commercial production at all. Y.K. park et al. wrote about such sensors; however, these sensors were produced by screen printing. In this way, they obtained a high pH sensitivity along with durability and an easier and cheaper manufacturing process. Dye 3 [57] and RhYK [58] mixtures were used in this study. They were introduced into a polyester fabric. The produced sensors had a reaction faster than 10 s, and characteristic color changes in alkaline and acidic conditions were visible even at low gas concentrations. The sensors showed resistance to mechanical factors and were characterized by durability and reversibility after washing and drying. They have also been confirmed to contain limited amounts of hazardous materials. The gas-detection performance was tested at various gas concentrations by using a home gas tester connected to a computerized color-matching system. The color intensity corresponding to the sensor response was calculated according to Kubelka–Munk theory—the K/S value. It can be determined from the reflectance of the surface at the maximum absorption wavelength. A gas emission was carried out in a concentration range of 1–100 ppm and the color change was measured every 10 s for 2 min. The detection rate was defined as the time required to reach a color value of five—when this value is five, observers can perceive distinct colors. When the Dye 3 sensor was exposed to NH_3_ gas, the color changed from yellow to brown in proportion to the concentration shown in Figure 3. The detection speed is set to less than 10 s [59].

**Figure 4:**
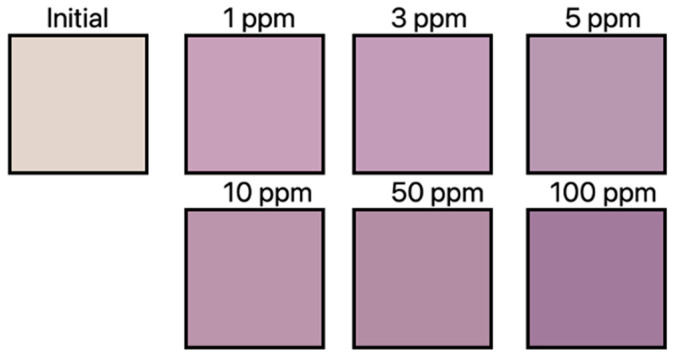
Sensor with RhYK color changes depending on HCl gas concentration.

After exposing the RhYK dye sensor to HCl gas, it changed its color in proportion to the concentration from colorless to purple, as shown in Figure 4. The detection speed was determined to be less than 10 s [59].

For both dyes, the initial increase in color values was faster with an increasing concentration of a given gas. This indicates that the detection rate increased with higher gas concentrations. A sensor made of a mixture of two dyes was also tested. The response of the mixed sensor to NH_3_ gas was the same as that of the sensor made of only one Dye 3. It also changed color from yellow to brown. The second RhYK dye did not react under the conditions tested. In the case of the HCl gas response, both dyes reacted. The color of the resulting sensor was reddish brown. The response in this case is much weaker than a sensor made of only one dye. A smaller amount of RhYK was used in the mixed sensor due to its poor solubility in ethanol. Despite the marked drop, the detection rate remained high at 10 s. Research work has shown that the solution can be used in protective clothing that changes its color under the influence of the presence of hazardous gases [59].

Another interesting project was the research of Y. K. Park et al., who presented an application of a colorimetric textile sensor fabricated by UV-induced photography for acid-gas detection based on halochromic dyes. Researchers synthesized two types of rhodamine graphable dyes by introducing a radical sensitive group into a rhodamine derivative with excellent pH sensitivity, and then textile sensors were fabricated by using UV photography. The produced sensors showed a color change in acidic conditions during testing. In addition, they were characterized by high durability, low energy consumption, no response to salts and alkalis and short process times. Scientists concluded that the sensor they made works properly and can find a wide range of applications and is also environmentally friendly [60].

The original sensor was praised by researchers led by D.Y. Cheong. They presented an electronic graphene fabric based on lotus fibers for gas detection. Graphene has become popular in textronic products and is widely used in them due to its properties being adequate for many applications. When manufacturing smart textiles, key parameters such as electrical conductivity, mechanical flexibility, weight and applicability to other practical applications should be kept in mind. The used lotus fiber has the appropriate properties for applications of textronic products based on graphene. Namely, the scientists gave the following parameters: lightness of about <1 mg, environmental friendliness, crease resistance, mechanical resistance and elasticity. In the study, the researchers developed a graphene-oxide-coated lotus fiber. Hydrogen interactions between the graphene flakes and cellulose fiber were used, which resulted in the required final product. It was found that with parameters such as a GO concentration of about 3 g/L and a fiber diameter of about 300 μm, the highest electrical conductivity was measured. The tested object showed a high value of electrical conductivity, the effective detection of dangerous NO_2_ gas molecules in a short exposure time, the detectability of small concentrations, selectivity and resistance to relative humidity, high mechanical flexibility and resistance to bending and stretching. The researchers said that the results showed that their product could be used as a wearable gas sensor [61].

Another textronic solution for detecting gases, in this case, ammonia, is twisted and made-to-measure V_2_O_5_/PANI/GO nanocomposite fabrics. X. Xing et al. proposed wearable NH_3_ sensors integrated into fabrics for real-time monitoring. Scientists first mixed graphene oxide with an aniline monomer and then polymerized it in situ with vanadium pentoxide to obtain ternary vanadium oxide, polyaniline and graphene oxide (KPW V_2_O_5_/PANI/GO). At room temperature, the sensor exhibited an enhanced response, high stability and resistance to mechanical factors such as twisting movements. Researchers determined that the described manufacturing method could be extended to nanosensing materials that could be integrated into other textiles [62].

L. Yanga et al. presented the design of a NO_2_ gas sensor based on silver laser-decorated graphene foam. In the production process, the researchers subjected a mixture of block copolymers and resin with different mass ratios to laser writing, which resulted in a highly porous laser graphene foam with various pore sizes. In addition, silver particles were added as a decoration to increase the surface properties and conductivity. The finished sensor is breathable, flexible, sensitive and selective. In addition, it is characterized by such features as a quick response and return to the initial state and a low level of detection of nitrogen oxides in the air. The finished sensor is fully suitable for use in textronic solutions, and it provides air circulation and moisture [63].

Another interesting project was the development by P. W. Oluwasanya et al. of nonencapsulated and washable two-dimensional electron textile material for detecting NO_2_ in ambient air. Researchers fabricated an electronic sensor on a nylon fabric resistant to standard washing cycles. They then carried out a coating with graphene oxide and GO/molybdenum disulfide and an in situ reduction of GO to reduced graphene oxide. The smart textiles made during the tests showed selectivity and high sensitivity in dry air; in moist air, the sensitivity was slightly worse but still at the appropriate level. The sensor showed resistance to washing. Researchers unanimously concluded that the textronic solution can be used in smart textiles for routine use [64].

A. K. Anbalagn et al., in their publication, presented a method of producing surface defects of zinc oxide nanorods by using gamma radiation to improve the detection properties of NO_2_ at room temperature. Scientists used a flexible paper substrate with polyethylene terephthalate by using a hydrothermal growing method to fabricate the zinc oxide nanorods. Surface modifications with gamma radiation were carried out by using a ^60^Co source. Researchers conducted detection experiments on prepared samples. It was found that at doses of 1.5 kGy, 4.5 kGy and 6.0 kGy, the responses were increased by 120%, 160% and 185%, respectively, at a concentration of 500 ppb, compared to the sensor response of 80%. Scientists have shown that surface modifications with gamma radiation affect the response of the sensor, improving its efficiency. The tests were carried out at room temperature [65].

## 5. Conclusions

So far, many publications have been published describing textronic solutions for gas sensors. Researchers from all over the world are looking for and analyzing many detection materials, the events that occur between the sensor and the gas and the possibility of detecting not one but many gases. Table 3 presents a summary of the above-mentioned works in terms of the parameters that determine the detectors.

## 6. Discussion

A safe house is not only one with a solid door or a proven antiburglary system. Carbon monoxide and natural gas or propane–butane sensors are also important. Carbon monoxide is called the silent killer for a reason. Even a small amount of poisoning can cause permanent damage to our health. One life motto is that it is better to prevent than to treat, so let us protect ourselves not only at home but also let us be vigilant against all types of harmful gas. The solutions in the article are designed to increase the safety of civilians and employees with high-risk professions. There are a lot of applications here.

All the textronic solutions described in this article are aimed at increasing safety or health monitoring, regardless of the purpose for which they were created—monitoring moisture, pH, biomolecules or harmful gases. They boast such features as correct operation, high sensitivity, stability and particularly reliable results of utility tests. However, there are some disadvantages to these solutions. Unfortunately, most of them are still in the research phase or have not yet been released for public use. Scientists are constantly working on minimizing textronic systems so that they are imperceptible to humans, but in some cases, the current technology does not allow it. It should be remembered that textronic systems mostly need a power source in the form of a battery.

The presented articles described various methods of production from many raw materials for many gases and only mentioned applications or created one prototype. Now, there is talk of putting sensors in clothing, but we do not have to stop there. It is especially important to support workers with high-risk occupations, but it is just as important to protect civilians. One can imagine smart curtains, net curtains or carpets that will change color under the influence of an increase in the concentration of a dangerous gas. When we let our imagination run further into the future, we can image household items that can alert emergency services when dangerous gases are present. Unfortunately, the existing publications only mention applications, and there is little information about the final products containing this technology.

There are many directions in which the presented technology can be successful. Depending on your needs, you can determine the speed of response, sensitivity or resignation from power sources of this technology. The ideal product would be one that would combine all these features. For this purpose, appropriate substrates, raw materials and methods of production or modification should be tested. The authors of this article set themselves the goal of making sensors for dangerous gases present in the everyday lives of those with high-risk professions or of ordinary people. The sensors will be developed both on textile substrates used to produce clothing and those used to produce household products, i.e., curtains, blinds or net curtains. As can be seen, the current methods are based on current flow and resistance changes. Efforts will be made to eliminate redundant electronic components or to minimize them as much as possible, and what is more, to make a prototype of the finished product.

As can be seen, all the described works had several variables in common. The detection substance had to be tested, produced or modified accordingly and combined into a system with a textile base. In the field of chemical sensors, there is a wide range of possibilities and undiscovered solutions. Existing studies should also be tested to detect more than one factor. You should ask yourself if a pH or moisture sensor is not able to be a detector of one of the gases.

## Figures and Tables

**Table 1:** List of values of the maximum permitted concentrations of selected chemical agents harmful to health in the working environment [54].

Harmful Chemical Agents	WEL * (mg/m^3^)	STEL **
Carbon dioxide	9000	27,000
Carbon monoxide	23	117
Sulfur dioxide	1.3	2.7
Ammonia	14	28
Chlorine	0.7	1.5
Hydrogen chloride	5	10
Hydrogen sulfide	7	14
Nitrogen oxides	2.5	-
Nitrogen dioxide	0.7	0.5
Propane	1800	-
Butane	1900	3000

* Workplace Exposure Limit; ** Short-Term Exposure Limit.

**Table 2:** Odor-detection threshold data for several harmful gases [55].

Name	Odor Detection Threshold (ppm)
Carbon dioxide	9.4
Carbon monoxide	100,000
Sulfur dioxide	1.1
Ammonia	5.2
Chlorine	0.31
Hydrogen chloride	0.77
Hydrogen sulfide	0.0081
Nitrogen dioxide	0.39
Propane–butane (odorized ethanethiol)	0.00035

**Table 3:** Gas-sensing performance in presented publications.

Ref.	Material	Gas	Concentration	Temperature (°C)	Response	Response/Recovery (s)
[56]	Graphene/ZnO nanorods on cotton fabric	CO	10–90 ppm	Room temperature	21.68–41.08%	370–435/45–115
[59]	Colorimetric textile based on halochromic dyes (Dye 3)	NH_3_	1–100 ppm	-	-	<10/-
[59]	Colorimetric textile based on halochromic dyes (RhYK)	HCl	1–100 ppm	-	-	<10/-
[60]	Colorimetric textile based on halochromic dyes (RhYK)	HCl	1–100 ppm	-	-	<10–20/-
[61]	Electronic graphene fabric based on lotus fibers	NO_2_	0–10 ppm	21	82%	-/-
[62]	V_2_O_5_/PANI/GO nanocomposites textile	NH_3_	0–10 ppm	24	31.2%	78/259 (1 ppm) 70/520 (20 ppm)
[63]	Silver laser-decorated graphene foam	NO_2_	0.5–2.5 ppm	-	12‰	40/291
[64]	GO and GO/MoS_2_ e-textile sensors	NO_2_	20–100 ppb	Room temperature	28%	-/-
[65]	Gamma-irradiated ZnO	NO_2_	0.5 ppm	25	185	-/-
[65]	Gamma-irradiated ZnO	NO_2_	1.5 ppm	25	312	-/-

## Data Availability

Data sharing not applicable. No new data were created or analyzed in this study. Data sharing is not applicable to this article.
